# p53 Target Gene SMAR1 Is Dysregulated in Breast Cancer: Its Role in Cancer Cell Migration and Invasion

**DOI:** 10.1371/journal.pone.0000660

**Published:** 2007-08-01

**Authors:** Kamini Singh, Devraj Mogare, Ramprasad Obula Giridharagopalan, Rajinikanth Gogiraju, Gopal Pande, Samit Chattopadhyay

**Affiliations:** 1 National Centre for Cell Science, Pune University, Pune, Maharastra, India; 2 Centre for Cellular and Molecular Biology, Hyderabad, Andhra Pradesh, India; Birmingham University, United Kingdom

## Abstract

Tumor suppressor SMAR1 interacts and stabilizes p53 through phosphorylation at its serine-15 residue. We show that SMAR1 transcription is regulated by p53 through its response element present in the SMAR1 promoter. Upon Doxorubicin induced DNA damage, acetylated p53 is recruited on SMAR1 promoter that allows activation of its transcription. Once SMAR1 is induced, cell cycle arrest is observed that is correlated to increased phospho-ser-15-p53 and decreased p53 acetylation. Further we demonstrate that SMAR1 expression is drastically reduced during advancement of human breast cancer. This was correlated with defective p53 expression in breast cancer where acetylated p53 is sequestered into the heterochromatin region and become inaccessible to activate SMAR1 promoter. In a recent report we have shown that SMAR1 represses Cyclin D1 transcription through recruitment of HDAC1 dependent repressor complex at the MAR site of Cyclin D1 promoter. Here we show that downmodulation of SMAR1 in high grade breast carcinoma is correlated with upregulated Cyclin D1 expression. We also established that SMAR1 inhibits tumor cell migration and metastases through inhibition of TGFβ signaling and its downstream target genes including *cutl1* and various focal adhesion molecules. Thus, we report that SMAR1 plays a central role in coordinating p53 and TGFβ pathways in human breast cancer.

## Introduction

Nuclear matrix and matrix binding proteins maintain chromatin architecture that is altered in cancer [1]. MAR (Matrix Attachment Region) binding proteins (MARBPs) like p53, Ku, PARP, SATB1, Cux/CDP are involved in regulation of various physiological processes that include cell cycle progression, DNA damage-repair, apoptosis etc. [2]. Among these MARBPs, p53 is frequently mutated in more than 50% human cancer patients [3]. Some of these specific mutations allow p53 to bind to MAR sequences with higher affinity, distort double strand DNA and thus affect transcription [4]. DNA damage and other stress induce p53 mediated cell cycle arrest, apoptosis and cellular senescence through post-translational modification of p53 like phosphorylation, acetylation, sumoylation etc. that play role in regulating the stability and transcriptional activity of p53 [5]–[7]. Whereas N-terminal phosphorylation is important for stabilization, C-terminal acetylation regulates the DNA binding properties of p53 by interfering with its nuclear import-export, degradation and tetramerization [8]. Dual acetylation of p53 at K373/382 is required for its transactivation function and transient or prolonged acetylation decides the cell fate towards either cell cycle arrest or apoptosis [9], [10]. Other cell cycle regulatory proteins include various Cyclins and Cyclin dependent kinase (cdk) complex that are aberrantly expressed in cancer. Among all Cyclins, Cyclin D1 expression is one of the hallmarks of breast cancer progression and is considered as a positive diagnostic marker [11], [12]. Various growth factors such as IGF I, IGF II, TGF-β, retinoic acid etc. induce Cyclin D1 expression [13]–[16]. Apart from these growth factors, oncogenic signals mediated by *Ras*, *Src*, *Stats* and *Erb2* that are involved in cellular transformation also activate Cyclin D1 [17], [13], [18], [19].

Tumor growth and its metastatic potential are decided by the growth factors available in the surrounding microenvironment. One of the major cytokines, TGFβ plays a dual role in breast cancer by regulating both growth inhibitory and pro-migratory signals in primary and advanced stages of breast cancer respectively, as decided by the extent of *Ras* activity [20]. TGFβ signaling involves family of stress-activated kinases to exhibit its effect [21]. Receptor activated Smad2 is phosphorylated upon EGF activation by PKC and ERK [22], [23]. Phosphorylated Smad2 then oligomerizes with Smad4 and translocates into the nucleus to further activate its target gene transcription [24], [25]. Tumor metastasis is further enhanced by increased expression of one of the major TGFβ target gene CUTL1 [26]. Cux/CDP/CUTL1 is another MAR binding protein, known to regulate mammary specific gene transcription and breast tumorigenesis [27]. CDP also regulate various developmental genes and affects cell growth and differentiation [28].

SMAR1 was identified as a MAR binding protein from T cell library through its direct binding to MARβ sequence at TCRβ locus and affect V(D)J recombination as observed in SMAR1 transgenic mice [29], [30]. SMAR1 also repress Eβ mediated transcription at TCRβ via its interaction with Cux/CDP [31]. Interestingly, SMAR1 shows more than 99% identity with its human counterpart BANP that has been precisely mapped to the locus 16q24 [32]. Loss of heterozygosity (LOH) at this locus is frequently reported in breast, colon and prostate cancer and is correlated to Cyclin D1 deregulation [33]–[35]. This again suggested that SMAR1 might have a role in breast tumorigenesis.

In a recent report we have shown that SMAR1 interacts with HDAC1 associated repressor complex at Cyclin D1 promoter and allows histone deacetylation to cause its transcriptional repression [36]. Earlier, we have shown that SMAR1 is down modulated in various transformed cell lines and can reduce the tumorigenic potential of B16F1 through p53 stabilization [37], [38]. However, the regulation and role of SMAR1 in cell proliferation, cell invasion and metastasis in human cancer patients is not known. Here we show that SMAR1 expression is drastically reduced in malignant Infiltrating Ductal Carcinomas (IDC) grade I, II and III. Further we could establish that SMAR1 stabilizes p53 and thus positively regulates p53 is itself transcriptionally regulated by p53.

Both in breast cancer cell lines and patient tissue samples decreased SMAR1 expression was correlated with defective p53 expression pattern and increased Cyclin D1 levels. Thus, we propose that mutant or defective p53 in breast cancer might result in compromised SMAR1 expression. Microarray analysis carried out in SMAR1 stable clone showed downregulation of various oncogenes, Cyclins, focal-adhesion molecules, TGFβ related genes and upregulation of various growth inhibitory, DNA repair and stress response genes. Interestingly, we found that SMAR1 overexpression leads to reduced TGFβ activation whereas SMAR1 expression was reduced upon recombinant TGFβ1 treatment. Further, stable expression of SMAR1 was shown to inhibit the metastatic potential of mouse melanoma cell line B16F1 *in-vivo*. Thus, we establish that SMAR1, another MAR binding protein, cross-talks between p53 and TGFβ signaling pathways and plays an important role in regulating tumor growth and metastases in breast cancer.

## Results

### SMAR1 is Downregulated in Breast Cancer

**Figure 1:**
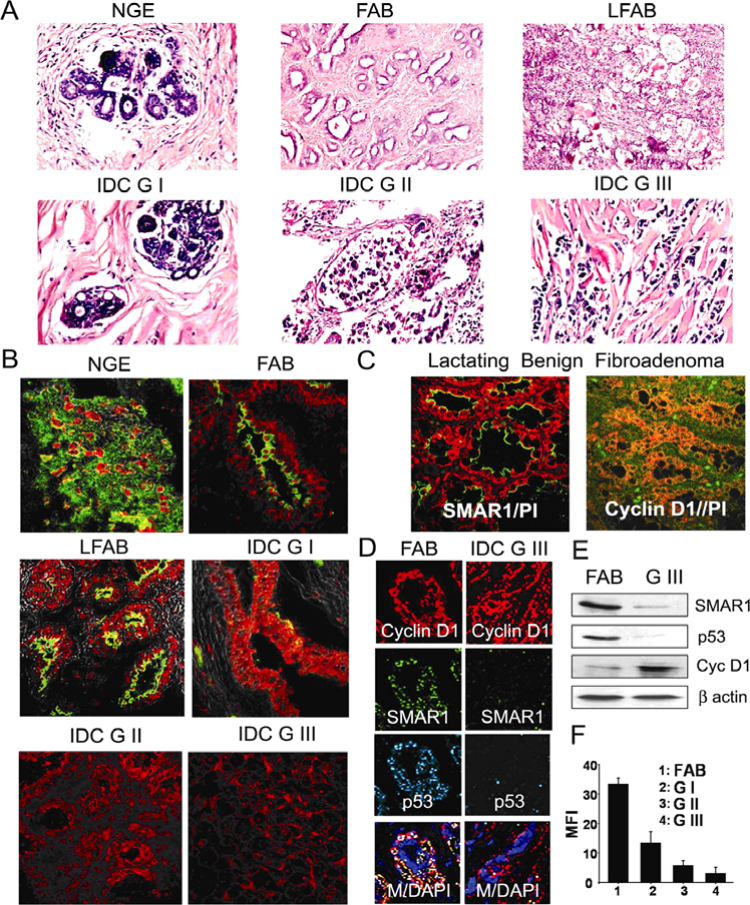
SMAR1 is downregulated during advancement of breast cancer. (A) Breast cancer tissue samples grading by HE staining into Normal Globular elements (NGE), Fibro-Adenoma Benign (FAB), Lactating Fibro-adenoma Benign (LFAB), Infiltrating Ductal Carcinoma Grade I- III (IDC G-I, II and III). (B) Immuno-fluorescence confocal staining for SMAR1 in NGE, FAB, IDC G I, II and III breast tumor sample using rabbit polyclonal α-SMAR1 probed with FITC conjugated α-rabbit secondary antibody shown as green, counterstained with propidium iodide to stain nucleus as red. (C) SMAR1 and Cyclin D1 expression using rabbit α-SMAR1 and rabbit α-Cyclin D1 probed with FITC conjugated α-rabbit secondary antibody shown as green, counterstained with propidium iodide to stain nucleus as red in Lactating Fibro-adenoma benign sample. (D) Expression analysis of SMAR1, Cyclin D1 and p53 in FAB and IDC G-III breast cancer sample by triple immuno-staining using rabbit α-SMAR1, mouse α-Cyclin D1 and goat α-p53 probed with donkey α-rabbit FITC, donkey α-mouse Cy3 and donkey α-goat Cy5 (Sky blue) respectively. Nucleus was counterstained by DAPI. (E) Representative Western blot showing the relative expression of SMAR1, Cyclin D1 and p53 in total protein lysate from fibro-adenoma (FAB) and IDC grade III tumor tissue sample. (F) Mean fluorescence intensity of SMAR1 staining in thirty FAB, ten IDC G-I, ten IDC-G II and ten IDC-GIII breast cancer samples were measured. Five different fields were taken for each sample and ±SD is indicated.

Human breast cancer samples were classified into fibro-adenoma (FAB), lactating benign fibro-adenoma (LFAB), Infiltrating Ductal Carcinoma Grade I, II and III (IDC G I, II and III) by standard HE staining. Thirty fibro-adenoma benign cases (including lactating benign fibro-adenoma) and thirty malignant cases (including grade I, II and III) were used for SMAR1 expression analysis. For comparison adjacent normal globular tissue area were taken as normal control. Nuclear polymorphism and hyperchromatasia was observed in all high-grade carcinoma (Figure 1A and 1B). SMAR1 expression was significantly reduced in all malignant IDC tissue samples as compared to either benign or normal (Figure 1B). Interestingly, the expression of SMAR1 was restricted to the cytoplasm in fibro-adenoma cases while it was present in both nucleus and cytoplasm as observed in adjacent normal tissue. SMAR1 expression was further correlated with Cyclin D1 levels where an inverse staining pattern was observed particularly in lactating benign fibro-adenoma. Whereas SMAR1 expression was observed in the outer layer of arrested epithelia, Cyclin D1 was expressed in all proliferating cells but not in the arrested epithelia (Figure 1C). SMAR1 was expressed in the early stages of breast cancer and showed colocalization with p53. Further, Cyclin D1 expression was higher in IDC grade III samples, while SMAR1 staining was drastically reduced (Figure 1D). Finally, Western blot analysis using whole protein lysate of breast cancer tissues confirmed the downregulation of SMAR1 and p53 along with upregulation of Cyclin D1 (Figure 1E). Further, quantitative analysis of SMAR1 staining in all breast cancer samples has revealed upto 2.5, 5.8 and 11 fold downregulation in mean fluorescence intensity (MFI) per 50 µm^2^ of the tissue area in IDC G-I, II and III as compared to the fibro-adenoma benign (Figure 1F). These results suggested that SMAR1 is downmodulated during breast cancer progression, which might be due to defective p53 function and as a consequence, Cyclin D1 expression is upregulated in the absence of its negative regulator SMAR1.

### SMAR1 Promoter Activity and Expression is p53 Dependent

**Figure 2:**
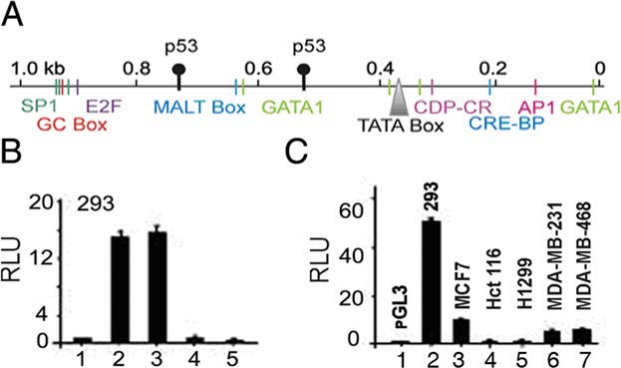
Identification and functional characterization of SMAR1 promoter. (A) SMAR1 promoter map showing various promoter elements with transcription factor binding sites including p53. (B) SMAR1 promoter luciferase assay in 293 cells showing promoter activity of 1.5 kb or 0.95 kb sense promoter constructs (lanes 2 and 3) respectively compared to the pGL3 vector control (lane 1). In addition 1.5 and 0.95 kb antisense SMAR1 promoter constructs were used as negative control (lanes 4 and 5). (C) SMAR1 promoter luciferase assay in 293 (lane 2), MCF7 (lane 3), H1299 (lane 4), Hct116 (lane 5), MDA-MB-231 (lane 6) and MDA-MB-468 (lane 7) compared to pGL3 basic vector control (lane 1). Error bars indicate standard deviation. Results shown are representative of at least five independent experiments.

Since, in advanced cases of breast cancer SMAR1 is deregulated, we wanted to delineate the mechanism of SMAR1 regulation. Gene map analysis showed that SMAR1 is located at human chromosome 16q24.3 locus. This region harbors tumor suppressor genes involved in breast cancers [33]. To further investigate the endogenous regulation of SMAR1 transcription, promoter region was identified and characterized using homology search tools and Clustal W multiple sequence alignment. 1.1 kb putative promoter sequence was identified and analyzed using Proscan. The TATA box is located at −655 along with MALT and GC box at −585 and −920 positions respectively. Transfac program predicted various transcription factor-binding sites including AP-1, GATA-1, CDP-CR, E2F etc. (Figure 2A). Along with these transcription factor-binding sites, SMAR1 promoter also harbors other response elements related to EGFR, TGFβ and Cyclin D1 (data not shown). Two fragments 0.95 kb and 1.5 kb immediate upstream of the ATG, were cloned into luciferase reporter system and promoter activities were analyzed. Upto 14 and 15 fold higher promoter activity was observed within 0.9 kb region (Figure 2B lane 2) and 1.5 kb promoter region (Figure 2B lane 3) respectively as compared to the control (Figure 2B lane 1) in 293 indicating that the maximum promoter activity resides within 0.95 kb region. The antisense clones of the same regions did not show remarkable promoter activity (Figure 2B lane 4 and 5). Interestingly, upstream of TATA box there are two putative p53 response elements present at −369 and −170 indicating a possible involvement of p53 in SMAR1 transcription (Figure 2A). To check this, both p53^−/−^ or mutant cell lines were used. Compared to WT p53 containing cell lines (293 and MCF7), p53 null cell lines (H1299 and Hct116) showed negligible promoter activity. Interestingly, 8–10 fold less promoter activity was observed both in MDA-MB-231 and MDA-MB-468 cell lines that express truncated and mutant p53 (lysine 273) respectively compared to 293 cells (Figure 2C lane 2, 6 and 7). Also there was 2–3 fold downregulation of SMAR1 promoter activity in MDA-MB-231 and MDA-MB-468 cell lines respectively as compared to MCF7 cells (Figure 2C lane 3, 6 and 7). These observations suggested that SMAR1 promoter activity is dependent on functional p53.

**Figure 3:**
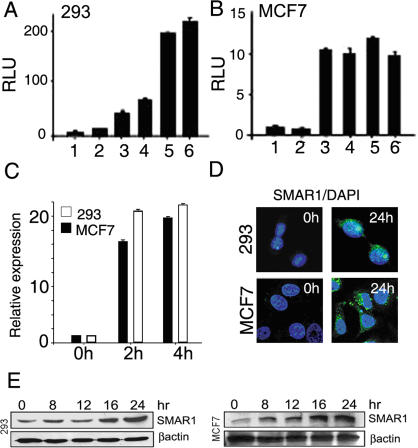
SMAR1 promoter activity is induced by Doxorubicin. (A and B) SMAR1 promoter luciferase activity upon 0.25, 0.5, 0.75 and 1.0 µM Doxorubicin (lane 3–6) compared to untreated cells (lane 2) and pGL3 basic vector control (lane 1) in 293 and MCF7 cell lines respectively. Relative Luciferase units are represented and error bars indicate standard deviation. Data is representative of three independent, simultaneously performed experiments. (C) Real time PCR analysis for SMAR1 in 0.5 µM Doxorubicin treated 293 and MCF7 cells respectively. Relative expression of SMAR1 transcript is shown and error bars indicate standard deviation. Data is representative of at least five independent experiments. (D and E) Immuno-fluorescence confocal analysis and Western blot analysis for SMAR1 in 0.5 µM Doxorubicin treated 293 and MCF7 cells. Scale bar represents 10 µm.

**Figure 4:**
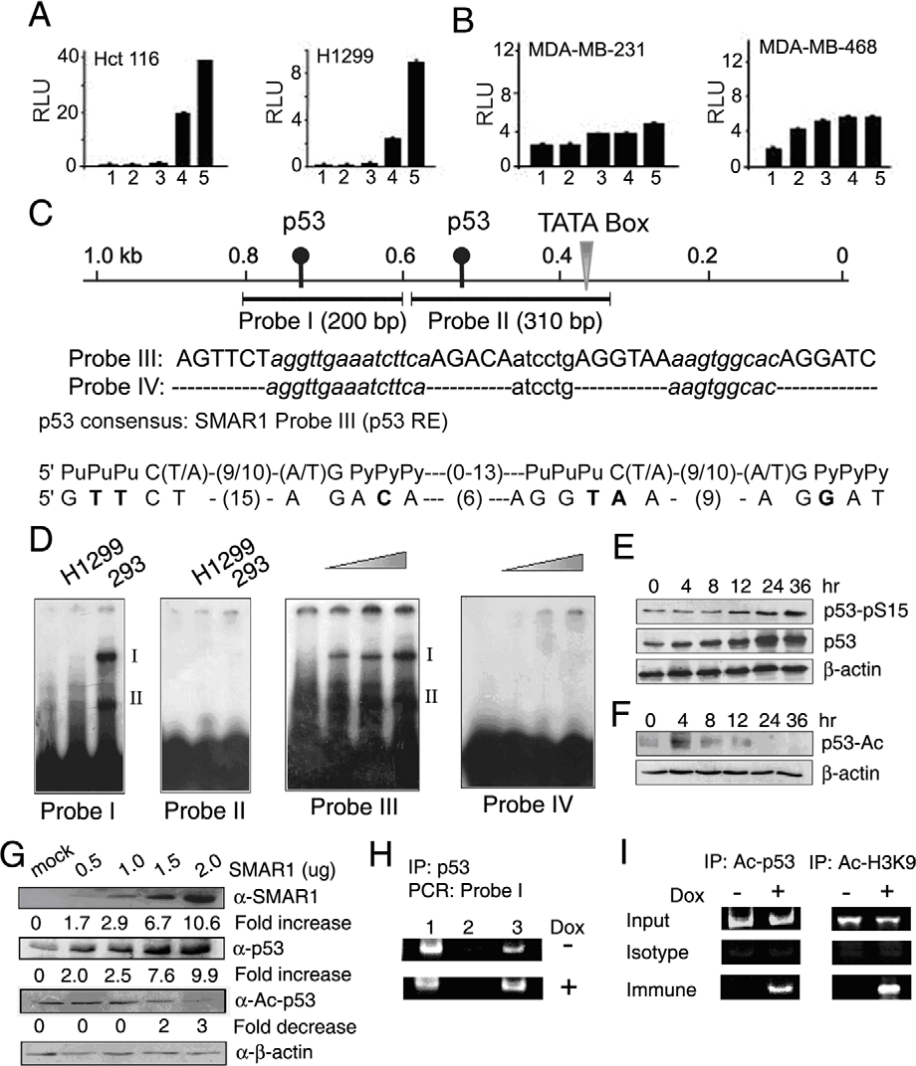
SMAR1 transcription is regulated by p53 through its direct recruitment on SMAR1 promoter. (A and B) SMAR1 promoter luciferase assay in H1299 (p53 null), Hct116 (p53 null) and MDA-MB-231 (p53 mutant), MDA-MB-468 (p53 mutant) cells (lane 2), SMAR1 promoter activity with 0.5 µM Doxorubicin (lane 3), along with co-transfection of WT p53 without and with 0.5 µM Doxorubicin treatment (lane 4 and 5 respectively) compared to pGL3 basic vector control (lane 1). Relative Luciferase units are represented along with error bars indicating standard deviation. Data is representative of three independent, simultaneously performed experiments. (C) Schematic representation of p53 binding site on SMAR1 promoter, sequence of WT p53 oligo (Probe III), deletion mutant oligo (Probe IV) and alignment of Probe III with p53 consensus binding site. Mismatches are given as bold. (D) EMSA using H1299 and 293 nuclear lysate showing p53 dependent complex-I only with probe I and not with Probe II (*first and second panel*). EMSA using 2.5, 5.0 and 10 µg of nuclear lysate from of 293 cells with probe-III and Probe IV (*third and fourth panel*). Complex-II came as non-specific signal. (E and F) Western blot analysis for total, phospho-serine-15 p53 and acetylated p53 K373/382 upon 0.5 µM Doxorubicin treatment from 0–24 hr in 293 cells. (G) Western blot analysis for acetylated p53 K373/382 upon increasing amount of pBK-CMV-SMAR1 transfection as compared to vector control in 293 cells. Relative fold change in expression is calculated by densitometric analysis normalized to the β-actin and are mentioned below respective panel. (H) Recruitment of p53 on the 200 bp region (Probe I) of SMAR1 promoter. Sonicated DNA fragments were pulled with α-p53 antibody and PCR for the 200 bp region was performed in the pulled DNA fragments. Lane 1 is the input PCR, lane 2 is the isotype (IgG) control and lane 3 is α-p53 pulled chromatin sample. (I) Recruitment of acetylated p53 and acetylated Histone-3 to the 200 bp region (Probe I) of SMAR1 promoter in the respective panels with and without Doxorubicin treatment. Input represent total sonicated genomic DNA, Isotype represents IgG control and Immune represents the pulled chromatin fraction by α-acetylated p53 and α-acetylated Histone-3 antibodies respectively.

Various stress signals causing DNA damage including many chemotherapeutic drugs like Doxorubicin, Camptothecin are known to activate p53 [39], [40]. To investigate the requirement of activated p53, Doxorubicin was used to induce p53 and SMAR1 promoter activity was assayed in various cancer cell lines. Doxorubicin treatment allowed induction of SMAR1 promoter activity by 5 fold in 293 and 4 fold in MCF7 cells respectively (Figure 3A and 3B). Further quantitative real time PCR analysis revealed that upon 4 hr of Doxorubicin treatment there was 18.7 and 19.5 fold increase in SMAR1 transcript in 293 and MCF7 cells respectively (Figure 3C). This further supported p53 dependent regulation of endogenous SMAR1 expression. Doxorubicin also induced SMAR1 protein expression as analyzed by confocal microscopy and western blotting in 293 and MCF7 cells respectively (Figure 3D and 3E). Similar results were observed in B16F1 cells (data not shown). Interestingly, Doxorubicin treatment failed to induce SMAR1 promoter activity both in p53^−/−^ and p53 mutant cell lines again confirming the requirement of active p53 for SMAR1 promoter function. To test if the N terminal phosphorylation of Serine residues can affect the promoter activity, two single amino acid p53 mutant constructs (ser15-ala and ser20-ala) were transfected in p53^−/−^ cell line. Both of the mutant p53 constructs failed to rescue SMAR1 promoter activity and its expression (data not shown). A significant restoration of promoter activity was observed upon introduction of WT p53 in the null cell lines H1299 and Hct116 (Figure 4A). Interestingly, WT p53 failed to rescue the promoter activity in MDA-MB-231 and MDA-MB-468 cells, expressing mutant p53 (Figure 4B). Also increasing dose of Doxorubicin did not induce endogenous SMAR1 expression in p53 null as well as mutant cell lines (data not shown). All These observations suggest that expression of WT p53 is required for SMAR1 gene expression.

### Recruitment of p53 at SMAR1 Promoter

Since SMAR1 promoter activity is dependent on p53 function, we further analyzed the p53 response elements present in SMAR1 promoter. The sequence analysis showed two putative consensus p53 binding sites at −369 and −170 present upstream of TATA box (Figure 4C). To further investigate the p53 binding at these response elements, two amplified probes of 200 bp (Probe I) and 310 bp (Probe II) corresponding to proximal and distal p53 REs were used for EMSA analysis. Binding assays were performed using nuclear lysate from 293 and H1299 cells. A specific p53 complex-I was found with Probe I using cell lysate that has WT p53 but not with the lysate from null cell line indicating that p53 directly binds to the probe I region. On the other hand, Probe II that includes another p53 binding site with shorter linker did not show p53 complex (Figure 4D). Distal p53 binding site was further minimized to 52-mer oligo (probe III) containing consensus p53 binding site and 32-mer (probe IV) deletion mutants, and were used as probe (Figure 4C). Sequence alignment of p53 RE (Probe III) on SMAR1 promoter showed significant identity with the consensus p53 binding site (Figure 4C). Upon adding increasing amount of nuclear lysate of 293 cells Probe III showed increased p53 complex, while probe IV failed to make any complex (Figure 4D). Probe III did not show any complex either with the nuclear lysate of p53 null cell lines (H1299, Hct116 and PC3) or with p53 mutant cell lines (MDA-MB-231 and MDA-MB-468) suggesting binding of WT but not mutant p53 (data not shown).

Since various post-translational modifications like phosphorylation, acetylation are known to affect p53 transactivation function, we checked the status of both phosphorylated and acetylated p53 in context to SMAR1 expression. While p53 expression was induced upto two fold in 4 hr, phospho-serine 15 was increased after 8 hr of Doxorubicin treatment in 293 cells (Figure 4E). This was then followed by increased SMAR1 transcription. Increased phospho-p53 after 8 hr indicates SMAR1 induction and concomitant stabilization of p53 due to SMAR1. This observation again confirmed our previous findings that SMAR1 overexpression leads to p53 stabilization in the nucleus [38]. We then checked the status of acetylated p53 upon Doxorubicin treatment and observed increased acetylated p53 at K373/382 residues within 4 hr that retained till 8 hr (Figure 4F). Since, acetylated p53 was reduced after 24 hr, we further checked whether SMAR1 could alter p53 acetylation. SMAR1 overexpression in 293 cells resulted in upto 3 fold decreased p53 acetylation (Figure 4G). To further investigate the direct binding of total as well as acetylated p53 on SMAR1 promoter, ChIP assays were performed. Both in Doxorubicin treated and untreated samples, total p53 were bound to 200 bp promoter region (Probe-I) of SMAR1 containing p53-binding site (Figure 4H). Interestingly, acetylated p53 at K373/382 was bound only in Doxorubicin treated samples (Figure 4I). Further using α-acetylated Histone-3 (K9), we found that in Doxorubicin treated samples, core Histone-3 was acetylated at lysine-9 (Figure 4I). Immunoprecipitation of all the proteins was confirmed by probing the chromatin pulled fraction with respective antibodies (Supplemetary Figure S1). Thus, recruitment of acetylated p53 on SMAR1 promoter in Doxorubicin treated samples resulted in its transcriptional activation through facilitating the core Histone acetylation at the locus.

Earlier, we have shown that overexpression of SMAR1 leads to cell cycle arrest [38], [36]. Since, Doxorubicin results in cell cycle arrest through activation of p53, we investigated the role of SMAR1 in Doxorubicin mediated cell cycle arrest. Doxorubicin treated 293 cells were subjected to FACS for cell cycle analysis. A shift of 9%, 13% and 15% cell population was observed towards G1/S phase after 8, 16 and 24 hr respectively in treated cells as compared to untreated cells. After 36 and 48 hr, Doxorubicin treated cells were arrested in G2/M phase. siRNA against SMAR1 along with Doxorubicin treatment, reduced the inhibitory effect of Doxorubicin on cell cycle, confirming the direct involvement of SMAR1 in Doxorubicin mediated cell cycle arrest (Supplementary Figure S2 and Supplementary Table S1). We further checked the status of various Cyclins like Cyclin D1, D3, A, E and found that all these Cyclins were downregulated whereas p27 level was increased upon Doxorubicin treatment. This downregulation was correlated with SMAR1 upregulation (Figure S3A). To further verify the contribution of SMAR1 in control of these cell cycle regulatory proteins, siRNA against SMAR1 was used in the presence of Doxorubicin. None of these Cyclins or p27 were affected by Doxorubicin in the absence of SMAR1 (Figure S3B). In summary, above results suggest that p53 acetylation caused by Doxorubicin activates SMAR1 promoter activity. The induced SMAR1 expression is then correlated with increased p53 phosphorylation at serine-15 and decreased acetylation at lysine 373/282. Thus, SMAR1 and p53 positively regulate each other where p53 activates SMAR1 transcription and SMAR1 stabilizes p53. Further cell cycle arrest at G1/S or G2/M phase is observed is correlated to induced SMAR1 expression and p53 stabilization. SMAR1 also cause reduction in p53 acetylation and hence limits p53 function to cause only cell cycle arrest and not apoptosis. In addition, SMAR1 also modulated the expression of other cell cycle regulatory proteins.

### SMAR1 Deregulation due to Altered Expression of Acetylated p53

**Figure 5:**
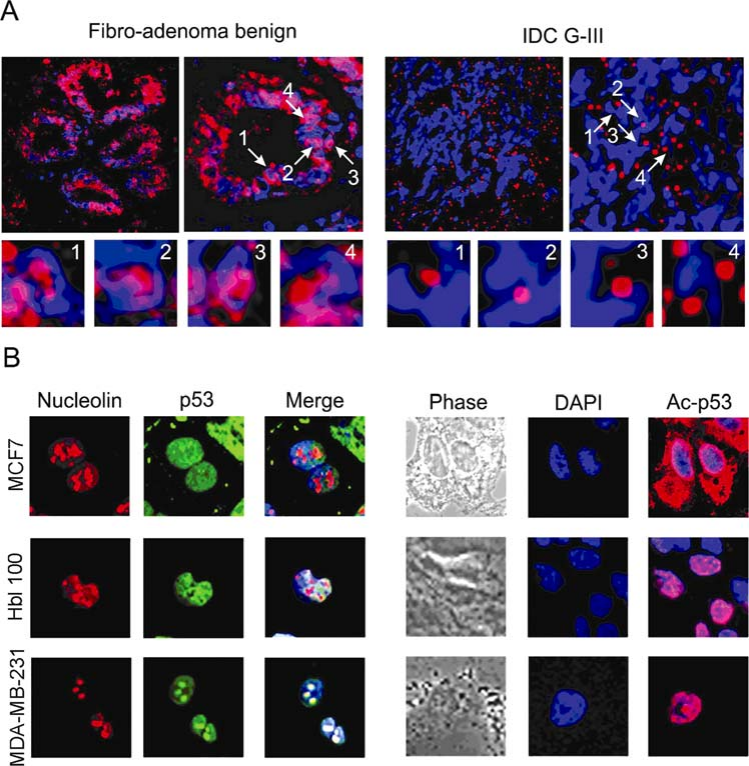
Localization of acetylated p53 in breast carcinoma. (A) Immuno-fluorescence confocal analysis for Ac-p53 in FAB and IDC G-III human breast cancer sample shown by using Cy3 conjugated α-rabbit secondary antibody (red) and nucleus is counterstained by using DAPI (blue). White arrows show accumulated acetylated p53 in the nucleolus (four respective magnified fields are shown as sub panels). (B) Immuno-fluorescence confocal analysis for p53, Ac-p53 and Nucleolin in breast cancer cell lines MCF7, Hbl-100 and MDA-MB-231.

Since SMAR1 is downregulated in advanced breast cancer samples and acetylated p53 is required for SMAR1 expression, we investigated the status of acetylated p53 in various grades of breast cancer samples by immuno-fluorescence. In benign tumor samples acetylated p53 was observed both in nucleus as well as in nucleolus whereas in the malignant samples, its levels were reduced and is completely sequestered into the nucleolus, compared to benign cases (Figure 5A). To support these results, we checked SMAR1, total p53 and acetylated p53 in various breast carcinoma cell line MCF7, Hbl-100 and MDA-MB-231 derived from breast epithelial fibro-adenoma, breast epithelial milk cell and IDC G-III respectively. Acetylated p53 at K373/382 was found both in cytoplasm and nucleus in MCF7 whereas nuclear and nucleolar expression were observed in Hbl-100 and MDA-MB-231 cells as shown by its co-localization with Nucleolin (Figure 5B). Thus, reduced SMAR1 expression in cell lines derived from IDC G-III sample can be attributed to the altered compartmentalization of mutant p53. Further, the phospho-serine-15 levels of p53 were drastically downregulated in benign and malignant stages of breast cancer (Figure S4A and B) implicating that once SMAR1 function is disrupted, p53 is no more stabilized.

### SMAR1 Inhibits Tumor Cell Migration and Invasion

**Figure 6:**
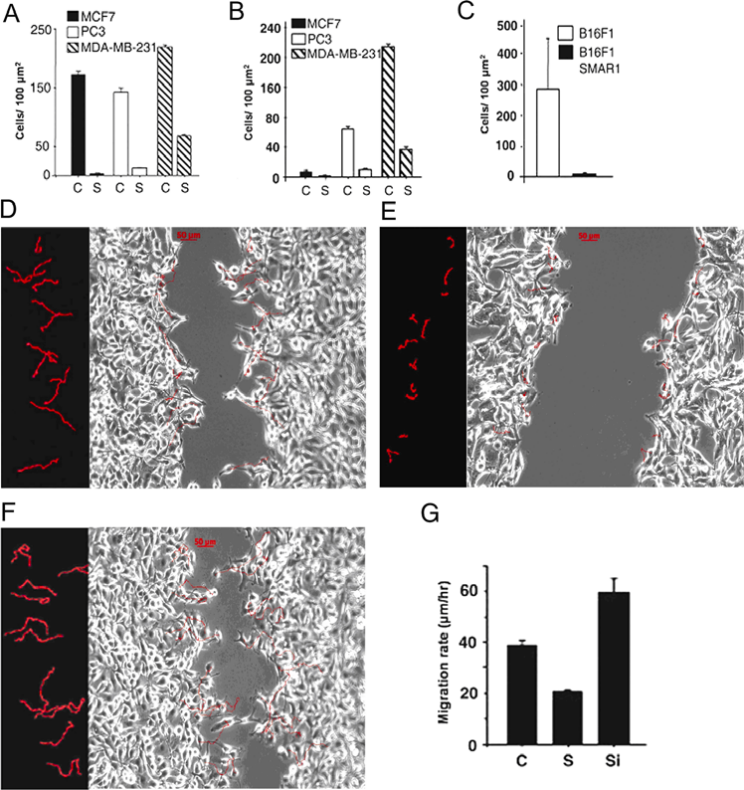
SMAR1 inhibits migration and invasion. (A and B) Two chamber migration and Matrigel invasion assay in MCF7, MDA-MB-231 and PC3 cells respectively. C refers to control cells and S refers to SMAR1 transfected cells. (C) Two-chamber invasion assay using SMAR1 stable and control B16F1 cells. Total number of cells were counted in five different fields and given as per 100 µm^2^ area. Error bars represent standard deviation and data is representative of three independent assays. (D) Time-Lapse image at the end of 10 hr in a wound-healing assay in control B16F1 cells. Few representative tracks are superimposed above the cells to show their movement during wound healing. The left panel of the image shows a selection of tracks of cell movement corresponding to the distance moved by the cells between 4^th^ hr and 10^th^ hr of time-lapse imaging. Bar 50 µΜ. (E and F) represent time-lapse images and the selection of the tracks for B16F1 cells stably transfected with SMAR1 and siRNA to SMAR1 respectively, during a wound-healing assay. The description of the image and the time points are similar to that of the control cells in (E). (G) Rate of migration of cells during the wound healing analyzed from the time-lapse microscopy of B16F1 control indicated as C, SMAR1 stable indicated as S and siRNA treated cells indicated as Si. Migration rate of at least 45 cells for each samples from five different fields were calculated. Error bars represent standard deviation and data is representative of three independent experiments.

To further examine the effect of SMAR1 on cell migration and invasion, we employed three different tissue culture assays that include wound healing assay, two chamber migration and two-chamber Matrigel invasion assays. Poorly and highly metastatic human breast cancer MCF7 and prostate cancer PC3 cell line were used. In wound-healing assay, we observed that SMAR1 transiently transfected cells poorly migrated compared to control cells (Figure S5A and S5B) Similarly, in two-chamber migration assay, MCF7, PC3 and MDA-MB-231 cells showed 50, 11 and 3 fold reduction in number of cells migrated respectively (Figure 6A). To further investigate the effect of SMAR1 on cell motility, Matrigel coated chambers were used and invasiveness of MCF7, PC3 and MDA-MB-231 cells were compared between vector transfected and SMAR1 transfected cells. SMAR1 overexpressing cells showed reduced invasion upto 3.5, 6 and 4.7 fold in MCF7, MDA-MB-231 and PC3 cell lines respectively (Figure 6B). Also SMAR1 stable clone in B16F1 mouse melanoma cells showed 17 fold reduced invasion compared to control B16F1 cells (Figure 6C). To further verify the inhibitory effect of SMAR1 in cell migration, we performed real time lapse video imaging assay in B16F1 SMAR1 stable clone and SMAR1 siRNA treated B16F1 and compared the migration velocity with respect to vector tranfected control B16F1 cells. Control B16F1 cells showed some movement towards the wound, though they did not fill the wound gap completely (Figure 6D). B16F1 cells stably transfected with SMAR1 showed only a little movement towards the wound and did not fill the wound gap at all seen in the picture and in the length of tracks (Figure 6E). B16F1 cells treated with siRNA for SMAR1 filled the gap considerably, showed higher motility and length of the cell tracks were longer compared to the control and SMAR1 stable cells (Figure 6F).

**Figure 7:**
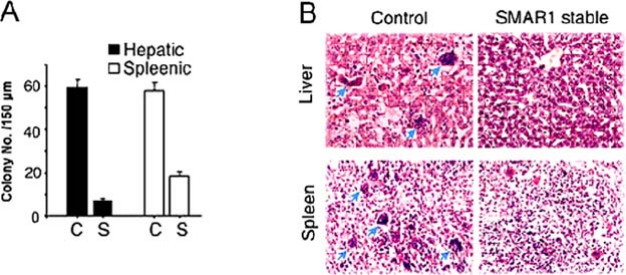
SMAR1 inhibits metastases in-vivo. (A) Quantification of the hepatic and splenic colonization obtained with SMAR1 stable indicated as S and B16F1 control cells indicated as C, 24 days post tail vein injection into nu/nu mice. Six nu/nu mice per sample were used for tail vein injection. Average number of colonies per animal as observed in serial H&E sections of liver and spleen at a distance of 150 µM from each other are represented along with ±SD of triplicate determinants. The assays were performed in triplicates. (B) Representative H&E staining showing hepatic and splenic colonization of control and SMAR1 stable B16F1. The arrows in (B) indicate the colonies of control B16F1 cells in liver and spleen. Images were taken at 20X magnification.

In the analysis of single cell migration in a wound healing assay SMAR1 stable clone showed almost 2 fold reduced and siRNA treated cell showed 1.5 fold increased migration rate as compared to the control cells (Figure 6G). For representative time-lapse video images see Supplemental Videos (Video S1, S2 and S3). To further verify the effect of SMAR1 expression in tumor metastases *in-vivo*, experimental metastases assay was performed in nude mice. Tail vein injection of SMAR1 stable and control cells showed 8.4 and 3.1 fold reduction in number of hepatic and splenic metastatic colony formation respectively (Figure 7A). Representative HE-stained tissue sections are given to show reduced colonization (Figure 7B). In summary, our results show that tumor suppressor SMAR1 causes inhibition of cell migration and invasion both *ex-vivo* and *in-vivo*. SMAR1 has been identified as a chromatin remodeling protein that controls gene expression by recruitment of other co-factors [36]. We therefore checked the modulation of specific genes by SMAR1 that are involved in migration and cellular metastases.

### SMAR1 Inhibits TGFβ Signaling and CUTL1 Expression

Transforming growth factor beta (TGFβ) has been earlier implicated to play a major role in tumor metastases by increasing expression of its downstream genes. Moreover, recently it has been depicted that, CUTL1, a target gene of TGFβ is highly expressed in advanced breast cancer and in turn enhances tumor cell metastases [26]. Since, SMAR1 is a global transcriptional repressor that represses various cell cycle regulatory genes like Cyclin D1, Cyclin D3, we suspected that SMAR1 might also affect the expression of either TGFβ or its downstream target gene CUTL1. Further a detailed microarray analysis performed in SMAR1 stable clone in B16F1 has shown significant downregulation in many oncogenes including *Ras*, *Myc*, *Stat1*, *Fosb* etc. Various MAP-kinase signaling molecules were also downregulated. Most of the Cyclins were reduced indicating the cell cycle inhibitory role of SMAR1. Interestingly, we could also see downregulation of TGFβ related molecules including important focal adhesion molecules like Vinculin, Fibronectin, ICAM5, Cadherin 3, Integrin α5 etc. which play major role in cell migration and motility whereas upregulation of tumor suppressor genes and genes involved in cell cycle and DNA metabolism (Tables 1 and 2). Most of these are TGFβ target genes, suggesting that SMAR1 might inhibit cellular migration and invasion by modulating TGFβ signaling.

**Table 1:** Genes downregulated by SMAR1.

Annotation	Fold change (log base 2)	Gene symbol	Description
**Cell cycle**			
NM_007628	1.5	Ccna1	cyclin A1
NM_017367	1.2	Ccni	cyclin I
NM_009833	1.0	Ccnt1	cyclin T1
NM_007635	1.1	Ccng2	cyclin G2
NM_007632	1.1	Ccnd3	cyclin D3
NM_133947	1.4	Numa1	nuclear mitotic apparatus protein 1
NM_009875	2.3	Cdkn1b	cyclin-dependent kinase inhibitor 1B (P27)
**Oncogenic signalling**			
NM_011785	2.1	Akt3	thymoma viral proto-oncogene 3
NM_008036	1.8	Fosb	FBJ osteosarcoma oncogene B
NM_011952	1.2	Mapk3	mitogen activated protein kinase 3
NM_008416	1.8	Junb	Jun-B oncogene
NM_010884	1.7	Ndrg1	N-myc downstream regulated gene 1
NM_013864	2.1	Ndrg2	N-myc downstream regulated gene 2
NM_016896	1.3	Map3k14	MAP kinase kinase kinase 14
NM_013931	1.3	Mapk8ip3	MAP kinase 8 interacting protein 3
NM_009101	2.0	Rras	Harvey rat sarcoma oncogene, subgroup R
NM_009283	1.6	Stat1	signal transducer and activator of transcription 1
NM_011305	2.7	Rxra	retinoid X receptor alpha
**TGFβ signalling**			
NM_009505	1.2	Vegfa	vascular endothelial growth factor A
NM_008542	1.0	Smad6	MAD homolog 6 (Drosophila)
NM_019919	1.4	Ltbp1	latent TGFβ binding protein 1
NM_025481	1.3	Smurf2	SMAD specific E3 ubiquitin protein ligase 2
**Adhesion**			
NM_010233	1.2	Fn1	fibronectin 1
NM_015799	1.7	Trfr2	transferrin receptor 2
NM_011926	1.5	Ceacam1	CEA-related cell adhesion molecule 1
NM_010875	3.1	Ncam1	neural cell adhesion molecule 1
NM_009502	1.2	Vcl	vinculin
NM_008319	1.5	Icam5	intercellular adhesion molecule 5, telencephalin
NM_010577	1.4	Itga5	integrin alpha 5 (fibronectin receptor alpha)
**Others**			
NM_019743	1.6	Rybp	RING1 and YY1 binding protein
NM_029083	1.5	Ddit4	DNA-damage-inducible transcript 4
NM_007602	2.1	Capn5	calpain 5

Genes downregulated in SMAR1 stable clone as compared to control B16F1.

**Table 2:** Genes upregulated by SMAR1.

Annotation	Fold change (log base 2)	Gene symbol	Description
**Cell cycle**			
NM_008613	1.4	Mns1	meiosis-specific nuclear structural protein 1
NM_013481	1.0	Bop1	block of proliferation 1
NM_007691	1.0	Chek1	checkpoint kinase 1 homolog (S. pombe)
NM_011045	1.4	Pcna	proliferating cell nuclear antigen
NM_018855	1.0	Gas8	growth arrest specific 8
**Tumor suppressor**			
NM_009765	1.2	Brca2	breast cancer 2
NM_134092	1.1	Mtbp	Mdm2, transformed 3T3 cell double minute p53BP
**DNA metabolism**			
NM_001013026	1.0	Ttf2	transcription termination factor, RNA polymerase II
NM_022811	1.0	Paf53	polymerase (RNA) I associated factor 1
XM_125902	1.2	Rex3	exportin, tRNA
NM_012012	1.8	Exo1	exonuclease 1
NM_011234	1.0	Rad51	RAD51 homolog (S. cerevisiae)
**Others**			
NM_008704	1.6	Nme1	expressed in non-metastatic cells 1, protein
NM_010494	1.0	Icam2	intercellular adhesion molecule 2
NM_023061	1.0	Mcam	melanoma cell adhesion molecule
NM_010722	1.0	Lmnb2	lamin B2
NM_013559	1.0	Hsp105	heat shock protein 105

Genes upregulated in SMAR1 stable clone as compared to control B16F1.

**Figure 8:**
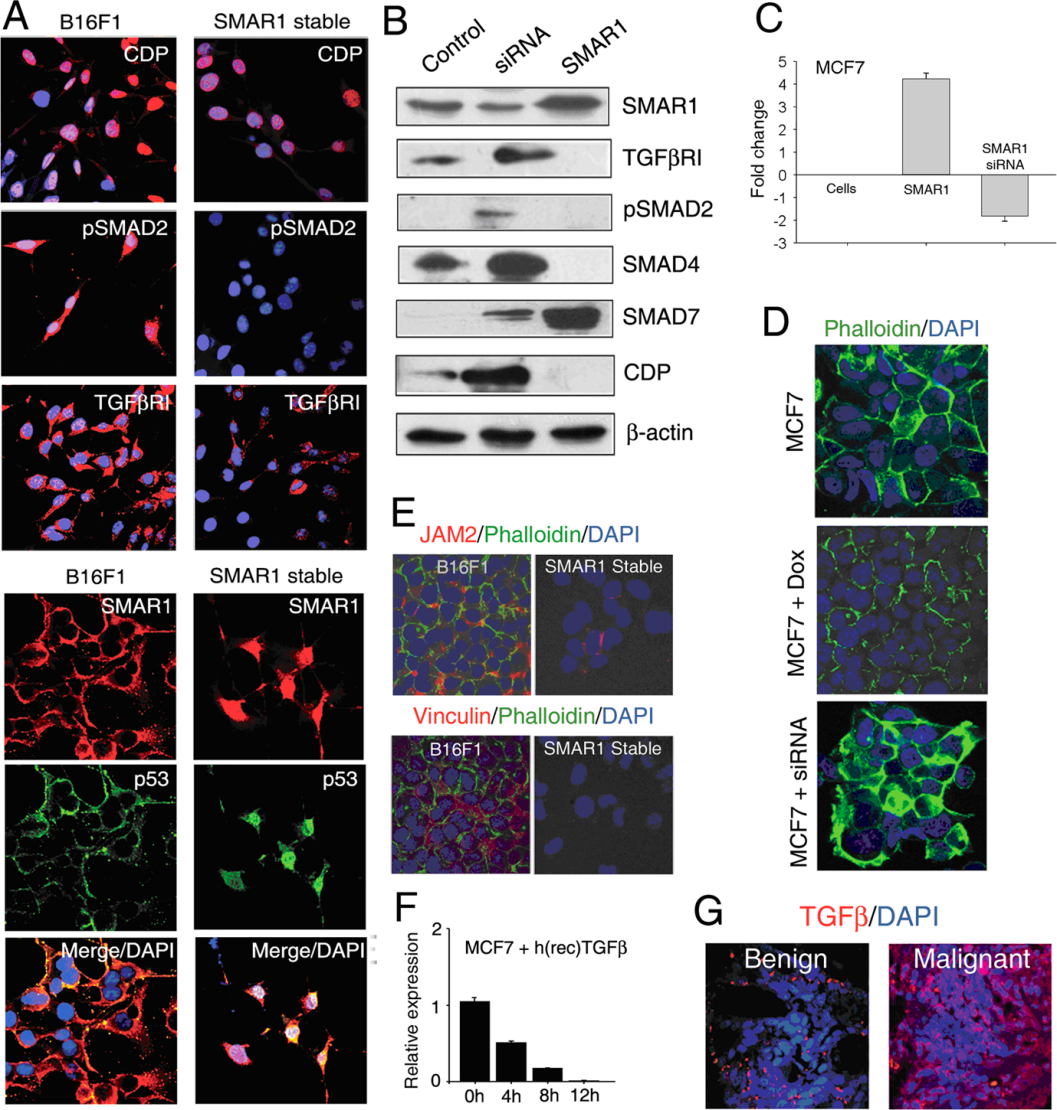
SMAR1 downregulates TGFβ signaling. (A) Immuno-fluorescence confocal analysis for CUTL1, TGFβ-R1, phospho-smad2, SMAR1 and p53 in SMAR1 stable clone and control B16F1 cells. (B) Western blot analysis for SMAR1, TGFβ-R1, phospho-smad2, Smad 4, Smad7 and CUTL1 in siRNA treated and SMAR1 transfected MCF7 cells compared to control cells. (C) Real time PCR for SMAR1 in MCF7 cells transfected with SMAR1 and in siRNA treated cells compared to control cells. Fold change are represented with ±SD of three independent experiments. (D) Immuno-flourescence confocal analysis for Vinculin and JAM2 in SMAR1 stable clone in B16F1 and control cells. (E) Alexa Fluor 488 phalloidin staining in MCF7 cells treated with Doxorubicin (0.5 µM) and siRNA for SMAR1 (100 nM) for 48 hr. (F) Real time PCR analysis for SMAR1 in MCF7 treated with human recombinant TGFβ1 (10 ng/ml) for 0–12 hr. Relative expression of SMAR1 is shown with ±SD of three independent experiments. (G) Immuno-fluorescence confocal analysis for secreted TGFβ1 in human benign and malignant breast cancer tissue sample.

Recently, CUTL1 another target gene of TGFβ, is shown to enhance tumor cell invasion and migration [26]. Thus, we checked the relative expression of CUTL1, phospho-Smad2, TGFβ-R1, SMAR1 and p53 by immuno-fluorescence in SMAR1 stable clone in B16F1 and control cells and observed a significant decrease in the expression of these proteins that correlated with SMAR1 and p53 expression (Figure 8A). Further, in MCF7 cells SMAR1 overexpression resulted in decreased TGFβ-RI, phospho-Smad2, Smad4 and increased Smad7 expression. Reversed effect was observed in siRNA treated sample (Figure 8B). Interestingly, we observed that CUTL1/CDP expression is drastically reduced upon SMAR1 overexpression and increased upon SMAR1 siRNA treatment (Figure 8B). SMAR1 expression was analyzed by real time PCR in GFP-SMAR1 and siRNA transfected cells where 4.5 fold upregulation and 1.5 fold downregulation of SMAR1 was observed in respective samples as compared to the mock-transfected MCF7 cells (Figure 8C). These results also corroborate with the earlier report that Smad7 controls cell-cell interaction and its overexpression inhibits breast cancer metastases [41]. Further we checked the effect of SMAR1 upon Vinculin, Fibronectin, Junctional adhesion molecule 2 (JAM2) expression and F-actin organization. Vinculin associates with focal adhesion and aderens junctions and promotes cell spreading and lamelllipodia formation [42], [43]. Fibronectin play vital role in maintaining the stability of extracellular fibrils and favors adhesion dependent cell growth and thereby enhance malignancy [44]–[46]. JAM2 a member of junctional adhesion molecules family expression also promotes cell migration [47], [48] Interestingly we found that the expression of Vinculin and JAM2 were decreased in SMAR1 stable clone in B16F1 cells as compared to the control cells (Figure 8D). Also in MCF7 cells treated with Doxorubicin for 48 hrs showed decreased expression of Vinculin, Fibronectin and JAM2 (data not shown). On the other hand SMAR1 siRNA treatment along with Doxorubicin resulted in increased expression of these molecules (data not shown). All these samples were also stained with Alexa Fluor 488 phalloidin to look into the F-actin organization to observe any cytoskeletal changes and found that the SMAR1 stable clone in B16F1 showed decreased F-actin expression (Figure 8D). Also Doxorubicin treated MCF7 cells showed round morphology instead of elongated with lowered F-actin expression as compared to the untreated cells and siRNA treated cells (Figure 8E). Since SMAR1 overexpression downmodulated TGFβ signaling and expression of its target gene CUTL1, we checked the reverse effect of TGFβ on SMAR1 expression. For this we treated MCF7 cells with human recombinant protein TGFβ1 and checked the expression of SMAR1 by real time PCR. We found drastic downregulation upto 50 fold in SMAR1 expression after 12 hrs of treatment (Figure 8F). Moreover a reverse correlation was found between SMAR1 and secreted TGFβ1 in benign and malignant breast cancer sample (Figure 8G). All these observations confirmed that SMAR1 negatively regulates cellular migration. Thus, decreased SMAR1 expression and activated TGFβ signaling in malignant breast cancer contributes to promote invasiveness of tumor cells.

## Discussion

**Figure 9:**
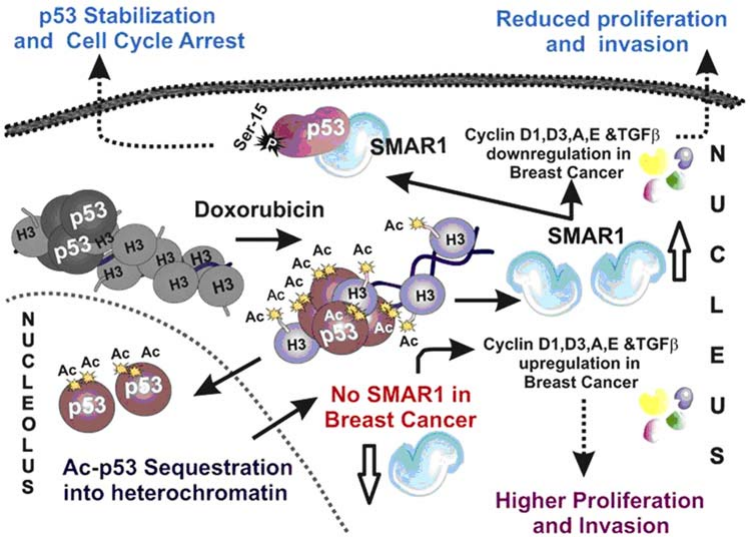
Model showing the regulation of SMAR1 by p53 upon Doxorubicin treatment and its implication in cell proliferation, migration and metastases.

MARBPs like CUTL1, SAF-A, SAF-B, p114, p53 etc. has been shown to alter chromatin integrity and nuclear matrix architecture that gets dysregulated in various cancers [1]. *Smar1*, located at 16q24.3 is a ubiquitously expressed MARBP. It is downregulated in many transformed cell lines including breast carcinoma cell lines MCF7, Hbl-100, MDA-MB-231, MDA-MB-468 etc. [36], [38]. Based on these findings we investigated the status of SMAR1 expression in human breast cancer patient samples. We found that SMAR1 expression is downmodulated in high-grade malignant human breast carcinomas compared to the benign samples. This was further correlated to the expression of Cyclin D1 that was increased in high-grade breast cancer. Since the downmodulation of SMAR1 in breast cancer was correlated to p53 and Cyclin D1 levels, we further investigated the mechanism of its regulation. To study this, we identified and characterized the promoter of SMAR1 located at human 16q24.3 locus. Along with other important transcription factor binding sites like GATA-1, E2F, AP-1, SP-1 etc. we identified two putative p53 binding motifs of which one was shown to be functional. SMAR1 has been shown to be involved in chromatin remodeling at TCRβ locus during V(D)J recombination [30]. Thus, we proposed that any stimulus that triggers chromatin changes might affect SMAR1 expression. Microarray analysis has also shown upregulation of DNA repair genes in SMAR1 stable clone again suggesting its direct involvement in processes related to DNA damage. Anticancer drug Doxorubicin was therefore used as a DNA damaging agent that acts through p53 activation and found that SMAR1 was induced upon Doxorubicin treatment in the cells containing WT but not mutant p53. Further WT p53 was shown to directly bind to its site present in SMAR1 promoter and induce its activity whereas mutant p53 failed to do so. Thus, while SMAR1 stabilizes p53 through direct interaction and phosphorylation, its own transcription is dependent on p53. Interestingly, we observed that Doxorubicin allows p53 acetylation, the onset of which triggers SMAR1 promoter firing (see model; Figure 9). In a time dependent Western blot analysis we found that upon Doxorubicin treatment, induction of p53 acetylation at K373/382 corresponds with SMAR1 induction. Chromatin immunoprecipitation assays showed that Doxorubicin induced DNA damage consequently leads to the recruitment of acetylated p53 on SMAR1 promoter and thus activate its transcription. Along with p53 acetylation, recruitment of p53 dependent activator complex on SMAR1 promoter also resulted in H3-K9 acetylation indicative of active chromatin at the locus. Once SMAR1 reaches a threshold expression, it allows deacetylation of p53 as shown upon SMAR1 overexpression. Upregulation of acetylated p53 is followed by induction in SMAR1 expression that is further correlated with the cell cycle where the SMAR1 expression was highest during G1/S phase. Prolonged treatment of Doxorubicin resulted in G2/M arrest, which can be explained due to SMAR1 mediated stabilization of p53 in the nucleus. siRNA treatment of SMAR1 inhibited Doxorubicin mediated cell cycle arrest. Cell cycle progression is thus regulated by p53 in response to DNA damage through regulation of SMAR1 expression that is directly involved in chromatin remodeling at the Cyclin D1 promoter loci. This suggests that SMAR1 functions in synergism with the Doxorubicin by conferring selective repressor activity to p53 through its deacetylation that results only in cell cycle arrest and not apoptosis. Thus, in addition to previously reported pathways [49] here we report a new positive feedback regulation between p53 and SMAR1 where SMAR1 is transcriptionally activated by p53 and that in turn stabilize p53 by facilitating phosphorylation at serine 15 residue.

The expression of SMAR1 is drastically downregulated during breast cancer progression and is inversely correlated with Cyclin D1 expression. Interestingly, we observed that acetylated p53 at K373/382 showed diffused expression in the nuclear spaces with higher expression in the nucleolar compartments in breast fibro-adenoma and exclusively nucleolar translocation in IDC G-III samples. These observations suggest that mutations in p53 may lead to its altered status and expression pattern in human breast carcinoma and is associated with its sequestration into heterochromatin resulting in its compromised activity. Similar correlation between p53 localization was observed in various breast carcinoma cell lines where p53 and acetylated p53 were predominantly present in the heterochromatin regions. All above observations suggest a possible mechanism of SMAR1 dysregulation in breast cancer due to abnormal p53 acetylation, phosphorylation and its sub-cellular sequestration.

Moreover, SMAR1 leads to reduced migration and invasion in both poorly and highly metastatic breast carcinoma cell lines irrespective of p53 status in both transient and stably SMAR1 transfected cells through downregulation of TGFβ signaling and its target gene expression including CUTL1. Earlier we have reported that SMAR1 inhibits ERK phosphorylation [37] that may contribute to decreased phospho-Smad2 levels and thus inhibits TGFβ signaling. SMAR1 is also able to reduce metastatic potential of B16F1 cells *in-vivo* in mice model. Microarray analysis done in SMAR1 stable clone, showing downregulation of various oncogenes, Cyclins, focal-adhesion molecules, TGFβ related genes and upregulation of various growth inhibitory, DNA repair and stress response genes also indicated anti-tumorigenic and anti-metastatic function of SMAR1. Our results establish SMAR1, as a critical regulator of TGFβ signaling cascade that finally affect CUTL1 expression. Downregulation of SMAR1 in high-grade breast carcinoma can be directly correlated with activated TGFβ signaling and its downstream target genes that consequently lead to increased tumor metastasis. Thus, we propose that SMAR1 acts as a key regulator of two major physiological processes of cellular proliferation and metastases in breast cancer by interplaying between p53 and TGFβ pathway and thereby prevent tumor cells to proliferate and metastasize (Figure 9).

In brief, we report SMAR1 as a transcriptional target of p53. Increased SMAR1 expression in turn results in p53 stabilization. Earlier PTEN-Akt, p14/19 ARF and Rb are reported to positively regulated p53 [49], here we report another protein SMAR1 that can also regulate p53 via positive feed forward loop. SMAR1 is downregulated in advanced breast carcinoma stages due to deregulated p53 function that again correlates with the elevated Cyclin D1 expression. Moreover, SMAR1 overexpression in both poorly and highly metastatic breast carcinoma cell lines leads to reduced migration and invasion irrespective of p53 status suggesting that the effect of SMAR1 in regulating genes involved in tumor migration and invasion is downstream of p53 although the wild type expression of SMAR1 is regulated by p53. Doxorubicin mediated upregulation of SMAR1 is p53 dependent and thus it does not function as efficiently in p53 mutated or null breast cancer cell line. Reduced migration and invasion observed in SMAR1 overexpressing cells were thus correlated to downregulated TGFβ signaling and inhibition of downstream kinase phosphorylation namely Smad2 phosphorylation. Further, various TGFβ target genes were observed to be downregulated such as *cutl1* that promote tumor cell metastases [26]. SMAR1 also inhibited the expression of Fibronectin, Vinculin and JAM2 that are involved in promoting cell-extracellular matrix adhesion, cell spreading and migration [42]–[48], suggesting that SMAR1 might prevent tumor cell metastases through negative regulation of these proteins. Thus, SMAR1 is an important anti-tumorigenic protein that regulates cell growth and metastases in breast cancer and acts as a connecting link between p53 and TGFβ pathway preventing tumor cells to proliferate and metastasize.

## Materials and Methods

### Mammalian cell culture and transfection

All cell lines were obtained from ATCC except Hct116 p53^−/−^ and H1299 p53^−/−^ cells that were obtained as kind gift from Dr. Kumar Somasundaram (Indian Institute of Science, Bangalore, India). Breast cancer cell line MCF7, Hbl100, mouse melanoma cell line B16F1, SMAR1 stable clone [38], Hct116 p53^−/−^, H1299 p53^−/−^ and human kidney embryonic cell line 293 were maintained in DMEM supplemented with 10% fetal calf serum (Invitrogen) in the presence of 5% CO_2_ at 37°C. MDA-MB-231, MDA-MB-468 cells were maintained in L15 media (Invitrogen) and PC3 cell lines were cultured Ham's F12 media (Invitrogen) supplemented with 10% fetal calf serum (Invitrogen) in presence of 5% CO_2_ at 37°C. Cells were seeded at a density of 1×10^6^ per 30 mm dish and cultured for 24 hr before transfection. One microgram of pEGFP-vector or 1 µg of pEGFP-SMAR1 plasmid DNA were used for transfection using Lipofectamine 2000, following manufacturers protocol (Invitrogen). For Luciferase promoter assays, SMAR1 promoter construct and pEGFP-p53 were used for transfection.

### Plasmids and siRNA

pBK-CMV-SMAR1 and GFP-SMAR1 expression constructs were used to overexpress SMAR1. Following siRNA specific for SMAR1 (Ambion, Austin, TX): 5′-UAACCCUGAGAUGCGGGUA with scrambled control RNA 5′-UACCGUAGGCAUGCAAUGG at 100 pM concentrations for 48 hr, with a pretreatment of 8 hr, was used to knock-down SMAR1 [37].

### SMAR1 promoter cloning and luciferase reporter assay

SMAR1 promoter sequence was identified and characterized using BLAST, Pro-Scan and Clustal W analysis. 950 bp promoter sequence was amplified from human genomic DNA using the following primer 5′-ATGATGTAGTTCCTGGGGTTTGA-3′ and 5′-CTGCGATAATGGCGTCCGTC-3′ (Genomechanix, USA) and was cloned in Luciferase reporter vector pGL3 basic via subcloning in pGMT-easy and pSP72 vector (Promega). Utilizing an internal *PvuII* restriction enzyme site orientation of promoter was checked and both sense and antisense constructs were selected for further experiments. SMAR1 promoter construct (0.5–2.0 µg/ml) was transfected with or without Doxorubicin (0.5 uM) treatment in p53 wild type cell lines. One microgram of GFP-p53, p53 Serine-15-Alanine and p53 Serine 20-Alanine plasmid constructs were used to express WT or single amino acid mutant p53 in p53 null cell lines with or without Doxorubicin (0.5 µM) treatment. Cells were harvested after 24 hr post transfection and/or treatment and were subjected to Dual Luciferase assay as per manufacturer's instruction (Promega). Luciferase activity was measured using Fluoroskan Ascent Luminometer (Lab Systems). All the assays were done in triplicates.

### Reverse Transcriptase and Real-Time PCR

2×10^5^ cells treated with Doxorubicin (0.2–1.0 µM; Sigma) or human recombinant TGFβ1 (10 ng/ml; Sigma) for 0–12 hours were harvested and total RNA isolated was subjected to cDNA synthesis following the manufacturer's instruction (Invitrogen). PCR was done using specific primers for SMAR1 F-5′-GCATTGAGGCCAAGCTGAAAGCTC-3′ and R-5′-CGGAGTTCAGGGTGATGAGTGTGAC-3′. β-actin was amplified using following primers F-5′-TACCACTGGCATCGTGATGGACT-3′ and R-5′-TTTCTGCATCCTGTCGGAAAT-3′, as a loading control. Real time RT-PCR was performed by icycler iQ thermal cycler system (Biorad) using double stranded DNA specific flurophore SYBR Green (BioRad) as per our published protocol [36]. Quantitation was performed with three different sets of cDNA samples. Graphs were plotted and statistical analysis was done using Sigma Plot.

### Immunoblotting and antibodies

Cells were scraped, washed with 1× PBS in different time intervals and lysed in DIGNAM buffer. Protein concentrations were estimated using Bradford reagent (Biorad). Equal amount of protein was loaded for immunoblotting. Following SDS-PAGE, resolved proteins were electroblotted on PVDF membrane (Amersham). The membrane was blocked overnight in TBS containing 0.1% Tween- 20 (TBST) and 10% BSA. The membrane was then probed with primary antibody in TBST for 2 hrs at RT or overnight at 4°C followed by three 10 min TBST washes at room temperature. Incubation with the secondary antibody was done for 1 hr, three 10 min TBST washes were given prior to chemiluminiscence detection using ECL substrate (Amersham). All antibodies for Western blots were obtained from Santa Cruz. Polyclonal rabbit antiserum was raised against recombinant GST-SMAR1 truncated (400–548 aa) fusion protein [37]. Mouse α-Cyclin D1, rabbit α-p27, TGFβ1, TGFβRI, phospho-Smad2 were procured from Cell Signaling and rabbit α-acetylated p53-K373/382 from Upstate Signaling. Secondary antibodies, goat α-mouse-HRP and goat α-rabbit HRP were purchased from Biorad.

### Immuno-flourescence

For Immunostaining 1×10^5^ cells were plated on coverslips and immunostained for SMAR1 using rabbit polyclonal α-SMAR1 and detected with secondary antibody mix containing FITC-conjugated α-rabbit IgG antibody (Bangalore Genei) following the standard protocol [37]. F-actin staining was done using Alexa Fluor 488 conjugated phalloidin (1U/ml; Molecular Probes, Invitrogen). Breast carcinoma tissues were obtained from KEM Hospital, Pune and Armed Force Medical College (AFMC, Pune). Histological grading of tumor tissues was done following modified Bloom and Richardson guidelines [50]. Immunoflourescence staining was carried out after de-parafinization by heating at 60°C for 5 minutes followed by partial rehydration in 100% and 95% ethanol. Antigen retrieval was done by boiling in 0.01 M citric acid. BSA blocked sections were stained with respective primary antibodies and detected by donkey α-Rabbit-FITC (Bangalore Genei), donkey α-Mouse Cy3 (Chemicon) and donkey α-Goat Cy5 (Chemicon) secondary antibodies. Nucleus was stained with Propidium Iodide (Sigma) or DAPI (Sigma). Sections and coverslides were mounted in anti-fade mountant medium (Sigma) and analyzed by Confocal laser microscope (LSM 510 version 2.01; Ziess, Thornwood, NY).

### Enzyme mobility shift assay

For EMSA, probes were PCR labeled using α^32^p dCTP in a 25 µl PCR reaction. For amplification of −369 to −569 (Probe I) primers used were (For 5′-TGCTGGGATTAAAGGTGTGC-3′, Rev 5′-CCTGTTTCCTGCCCGTTCCC-3′) and −170 to −480 (Probe II) primers used were (For 5′-GGGAACGGGCAGGAAACAG-3′, Rev 5′-TTCCGGGCTCGTTCAGTGGC-3′). PCR products were then eluted from native polyacrylamide gel by phenol-chloroform method and subsequent precipitation by 70% ethanol. Oligonucleotide labeling was done by klenowing reaction using α^32^ p dCTP in a 20 µl reaction containing 25 µM dATG mix, Klenow buffer and 0.5 U of Klenow (Invitrogen). Probe purification was done using Probequant G 50 column (Amersham). Binding reactions were performed in a 10 µl total volume containing 10 mM Tris (pH 7.5), 1 mM DTT, 50 mM KCl, 50 mM NaCl, 5 mM MgCl_2_, 5% glycerol, 1 µg double stranded poly (dI-dC), 10 µg BSA and 10 µg of nuclear lysate. Samples were incubated for 5 min at room temperature prior to addition of radiolabelled probe. Then the samples were incubated for 15 min at room temperature, the products of binding reactions were resolved by 8% native polyacrylamide gel electrophoresis. The gels were dried under vacuum and processed for autoradiography.

### Chromatin immunoprecipitation assays

Chromatin immunoprecipitation (ChIP) assays were performed using Chromatin Immunoprecipitation assay kit (Upstate Biotechnology) following manufacturer's instructions. 1×10^6^ cells were plated per 30 mm dish and treated with 0.5 uM Doxorubicin for 6 hrs. After treatments, DNA-protein interactions were fixed with 1% formaldehyde at 37°C for 10 min. ChIP assays were carried out using α-p53, (Santa Cruz) α-Ac-p53-K372/383 (Upstate Signaling) and α-H3K9 (Cell Signaling) antibodies. Input DNA, Rabbit IgG (r-IgG), and Mouse IgG (m-IgG) pulled DNA served as controls for all the experiments. DNA immunoprecipitated was then subjected to 35 cycles of PCR using primers for probe I. Amplified PCR products were analysed by native PAGE (10% Poly acrylamide gel) and ethidium bromide staining.

### Wound healing, migration and invasion assays

An artificial wound was made using a 10 µl pipette tip on confluent cell monolayers of vector or GFP-SMAR1 transfected cells, after 8 hr serum starvation and cell migration was observed in serum containing medium. Images were taken using Motorized IX-81 inverted microscope attached with DP70 CCD camera (Olympus). The number of cells migrated towards the wound was calculated per 10^5^ µm^2^ wound area after 12 hr. Cell migration and invasion were determined by using the modified two-chamber migration/invasion assay (8 µm pore size membrane uncoated/coated with Matrigel, BD Biosciences) according to the manufacture's instructions. 2×10^5^ cells were seeded in serum free media on the upper migration/invasion chamber and were incubated in the lower chamber containing media supplemented with 10% serum for 24 hrs in humidified tissue culture incubator, at 37°C, 5% CO_2_. After 24 hrs, cells from the upper chamber were removed by scrubbing and the migrated/invaded cells in the lower chamber were fixed and stained with 100% methanol and 1% Toluidine Blue respectively. Quantification was performed by counting the number of stained cells per 100 µm^2^ area migrated/invaded to the lower chamber.

### Time-lapse video microscopy

Time-lapse imaging of migrating cells in wound healing assay was performed on an Axiovert 200M microscope (Carl Zeiss, Germany) over 10 hr in serum containing medium in humidified chamber at 37°C and 5% CO_2_ atmosphere. Images were obtained every 2 min using a 10× phase objective of NA 0.25 and analyzed using image analysis software Metamorph Universal Imaging,USA. A minimum of 45 cells per sample were tracked to get the total distance traveled over a time period of 10 hr time period. The average migration speed in µm/hr was calculated and graphs were plotted using Microsoft Excel and Sigma plot program.

### *In-vivo* metastases assay

Male MNRI nu/nu mice (6 mice/group) were injected with 10^6^ B16F1 control or B16F1 stably expressing SMAR1 cells/0.1 ml PBS into the tail vain. Mice were sacrificed after 24 days post injection and serial sections of the liver and spleen cut at the distance of 150 µm from each other were hematoxylin-eosin (HE) stained. The number of hepatic and splenic colonies was counted in 15 sections per liver and spleen.

### Microarray analysis

Microarray experiment was commercially done by Agilant Genotypic Technology, Bangalore, India. Significantly regulated genes are presented with the fold >0.5 for downregulation and >1 for upregulation between the mean expression values of B16F1 control and SMAR1 stable clones in triplicate experiments.

## Supporting Information

Figure S1 Western blot analysis for total p53, Ac-p53 (K372/383), Ac-Histone 3 (K9) in chromatin immunoprecipitated fractions of Doxorubicin treated and untreated 293 cell lysate showing the respective immunoprecipitated proteins.
(8.88 MB TIF)

Figure S2 Cell cycle analysis by FACS. 293 cells were treated with Doxorubicin with or without SMAR1 siRNA for 0–48 hrs as mentioned in the figure. Percent population in G1, S and G2 phase are represented as M1, M2 and M3 markers. The result shown is representative of five independent experiments.
(6.00 MB TIF)

Figure S3 Western blot analysis for Cyclin D3, p27, Cyclin D1, Cyclin E, Cyclin A with β-actin as loading control in synchronized 293 cells upon 0.5 µM treatment of Doxorubicin with or with out SMAR1 siRNA (100 nM) after various time points corresponding to the FACS samples(A and B).
(10.44 MB TIF)

Figure S4 Immuno-fluorescence confocal analysis for phosphor-serine 15 p53 using rabbit polyclonal α-p53 ser-15 primary antibody and was detected by goat α-rabbit-Cy3 secondary antibody in Fibroadenoma (A) and Infiltrating Ductal Carcinoma grade III (B) breast cancer sample. DAPI was used to counter stain nucleus.
(7.71 MB TIF)

Figure S5 Wound healing assay in control MCF7 (A) and PC3 cells (B) or in cells transiently transfected with SMAR1. Images represent control cells and SMAR1 siRNA transfected cells at 0 hr and after 24 hr of transfection.
(7.66 MB TIF)

Table S1 Percent population shift towards G1/S and G2/M phase in Doxorubicin (0.5 µM) treated with and without siRNA (100 nM) compared to control untreated synchronized 293 cells.
(0.03 MB DOC)

Video S1 Time lapse video showing migration of control B16F1 cells.
(1.61 MB MOV)

Video S2 Time lapse video showing migration of SMAR1 stable B16F1 cells.
(1.62 MB MOV)

Video S3 Time lapse video showing migration of SMAR1 siRNA treated B16F1 cells.
(1.60 MB MOV)
