# Supplementary figures and images for: p53 Target Gene SMAR1 Is Dysregulated in Breast Cancer: Its Role in Cancer Cell Migration and Invasion
Source: PLoS One. 2007 Aug 1;2(8):e660. doi: 10.1371/journal.pone.0000660 (PMC1924604; doi:10.1371/journal.pone.0000660)

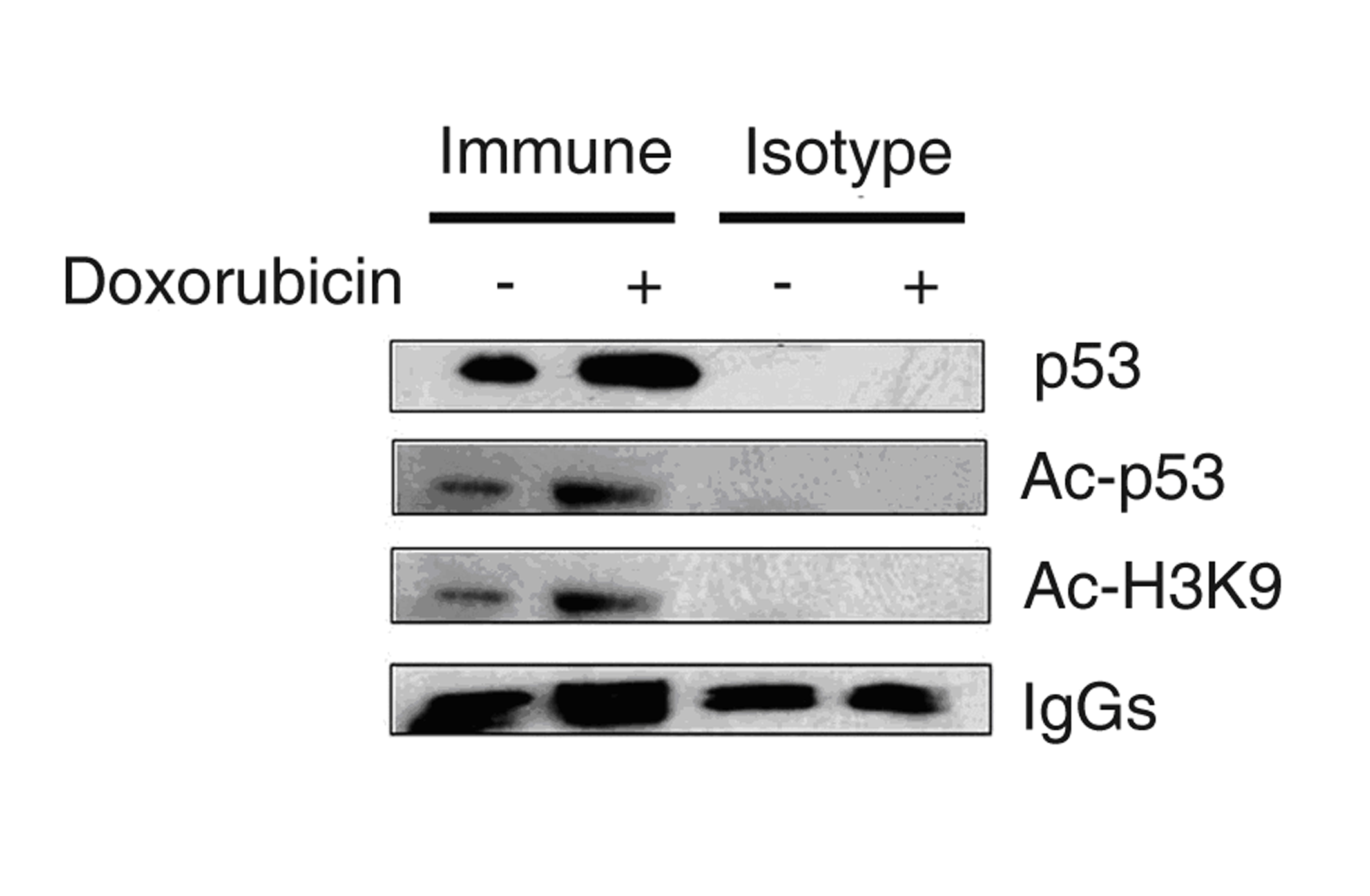

Supplement: Figure S1 — Western blot analysis for total p53, Ac-p53 (K372/383), Ac-Histone 3 (K9) in chromatin immunoprecipitated fractions of Doxorubicin treated and untreated 293 cell lysate showing the respective immunoprecipitated proteins. (8.88 MB TIF) [file pone.0000660.s001.tif]

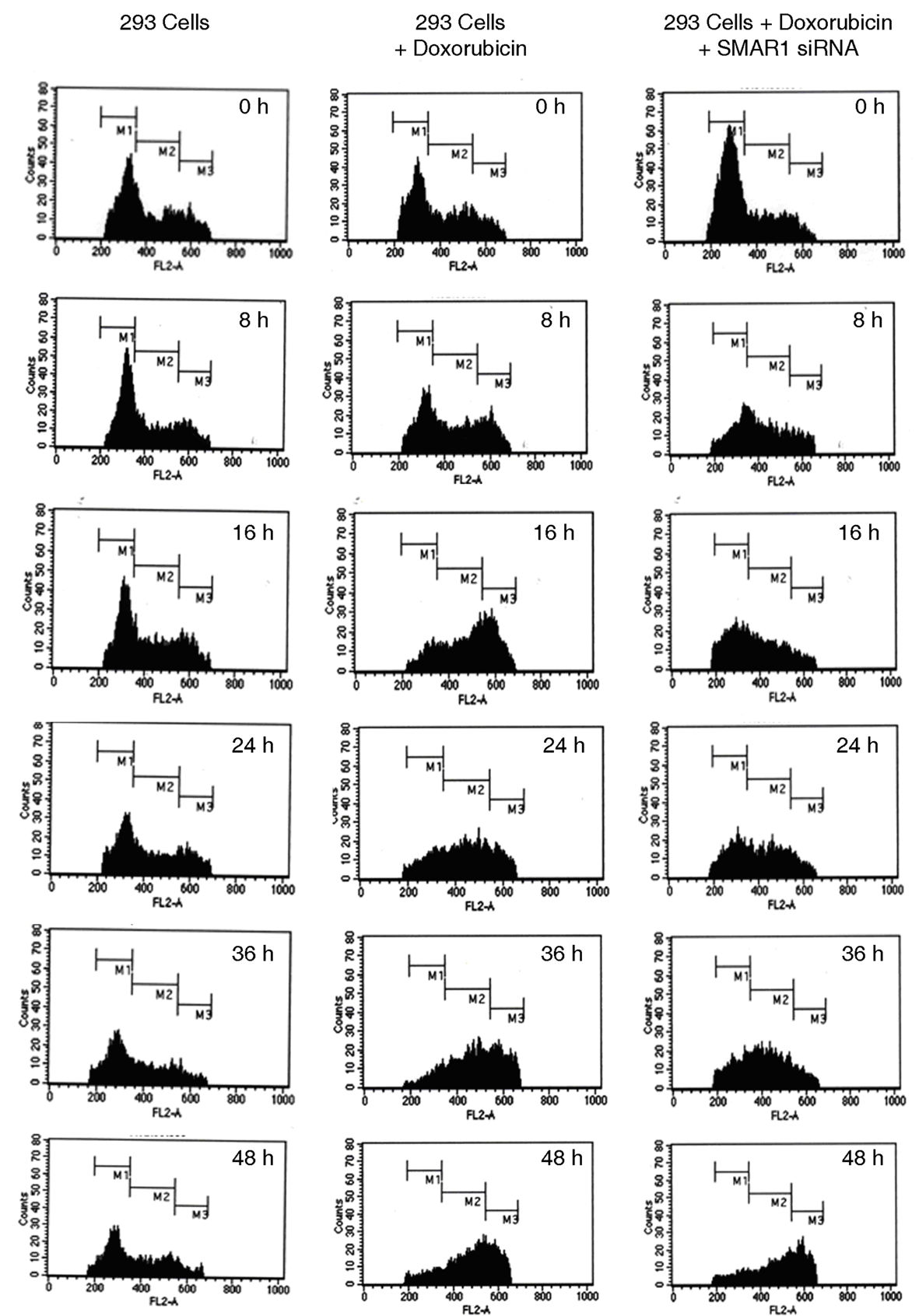

Supplement: Figure S2 — Cell cycle analysis by FACS. 293 cells were treated with Doxorubicin with or without SMAR1 siRNA for 0–48 hrs as mentioned in the figure. Percent population in G1, S and G2 phase are represented as M1, M2 and M3 markers. The result shown is representative of five independent experiments. (6.00 MB TIF) [file pone.0000660.s002.tif]

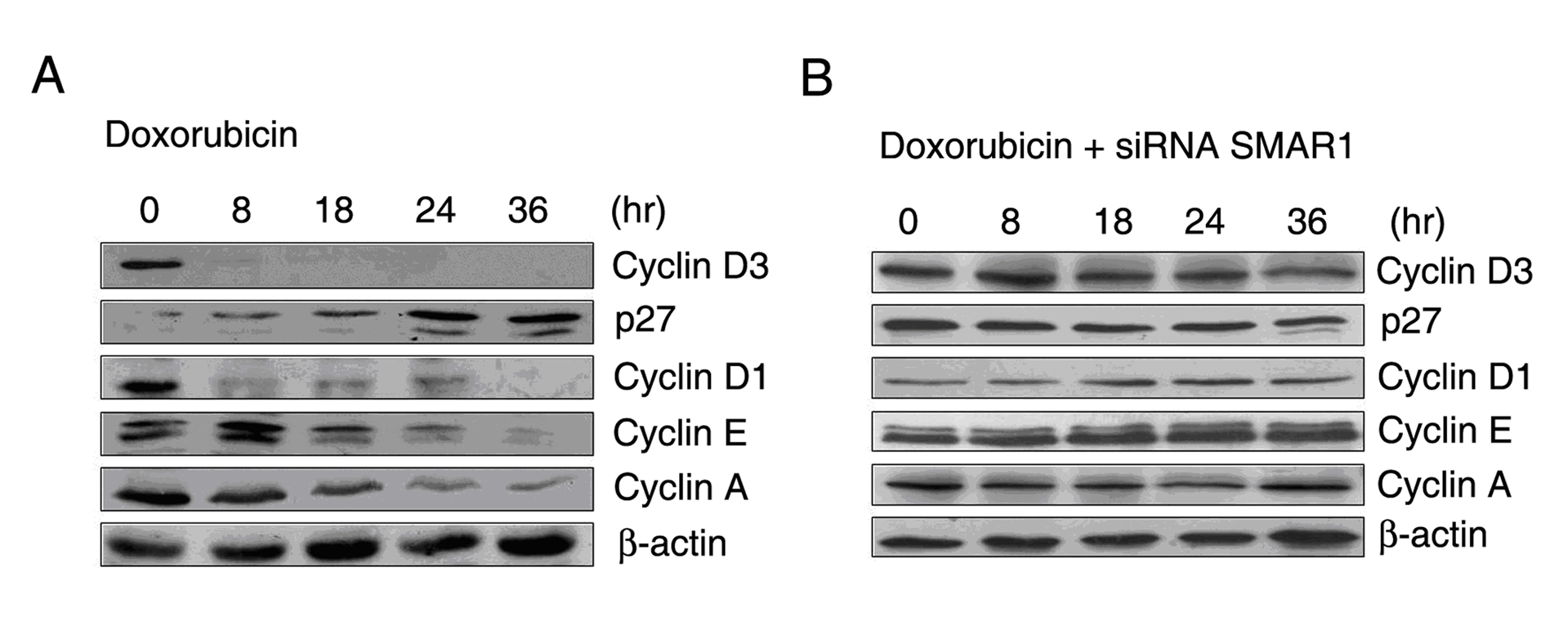

Supplement: Figure S3 — Western blot analysis for Cyclin D3, p27, Cyclin D1, Cyclin E, Cyclin A with β-actin as loading control in synchronized 293 cells upon 0.5 µM treatment of Doxorubicin with or with out SMAR1 siRNA (100 nM) after various time points corresponding to the FACS samples(A and B). (10.44 MB TIF) [file pone.0000660.s003.tif]

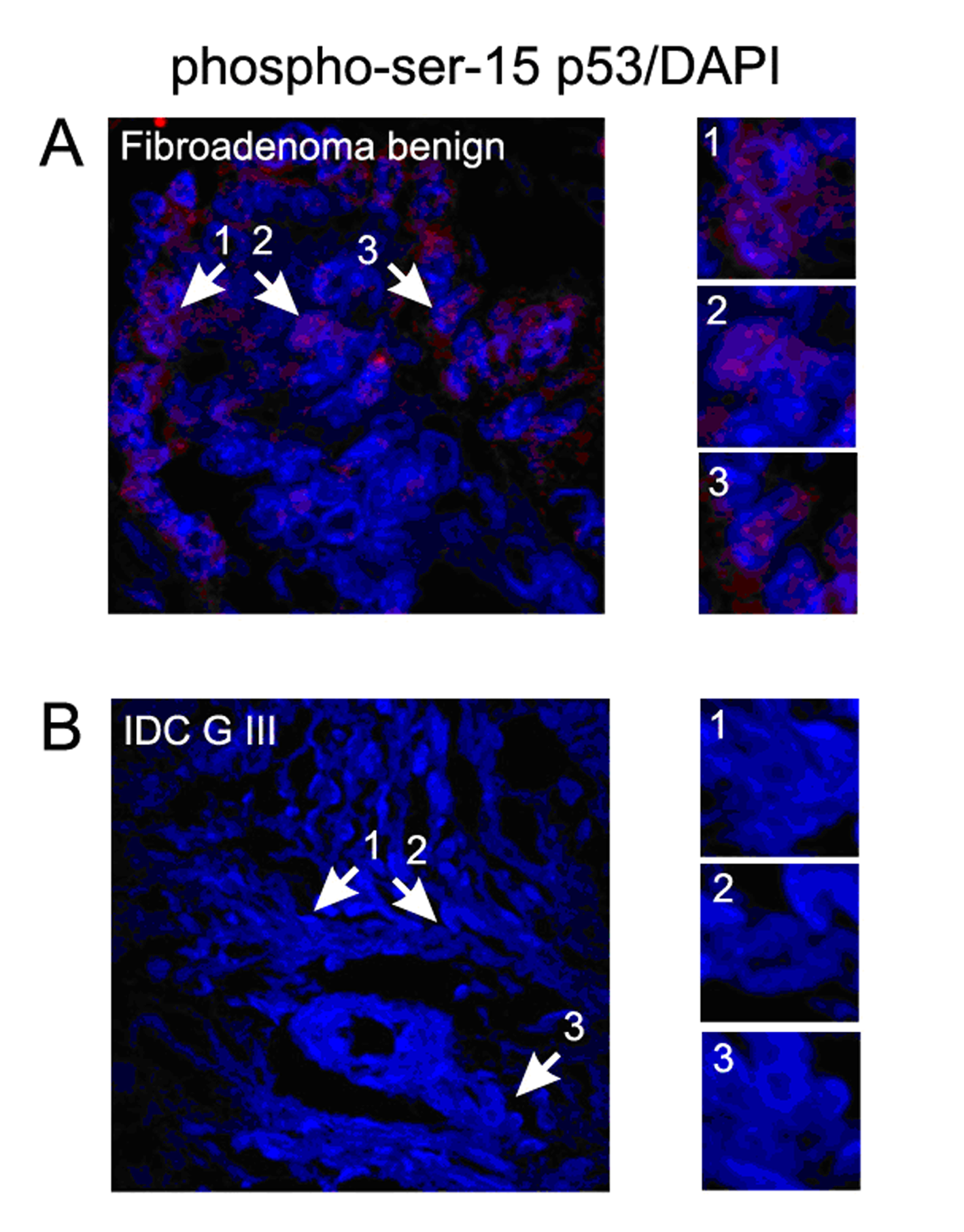

Supplement: Figure S4 — Immuno-fluorescence confocal analysis for phosphor-serine 15 p53 using rabbit polyclonal α-p53 ser-15 primary antibody and was detected by goat α-rabbit-Cy3 secondary antibody in Fibroadenoma (A) and Infiltrating Ductal Carcinoma grade III (B) breast cancer sample. DAPI was used to counter stain nucleus. (7.71 MB TIF) [file pone.0000660.s004.tif]

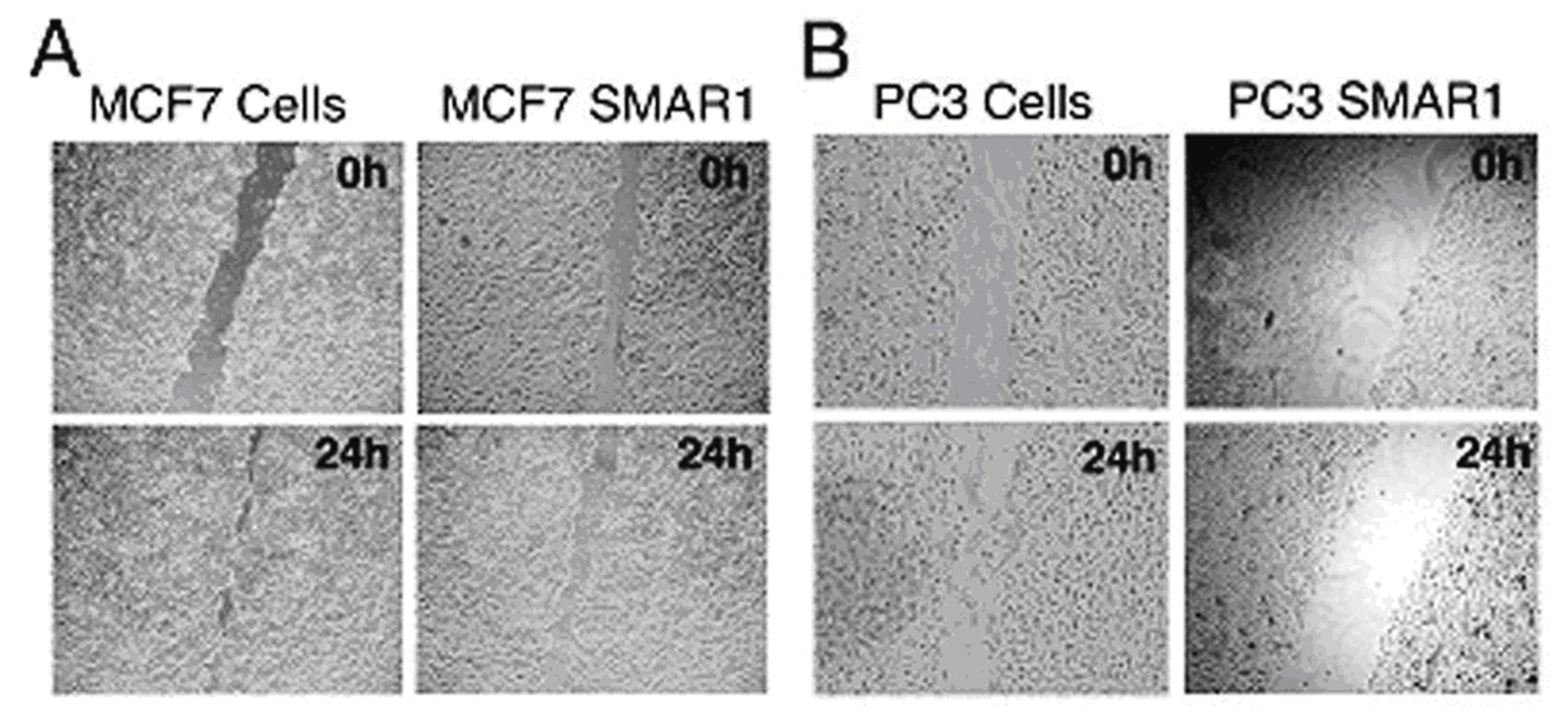

Supplement: Figure S5 — Wound healing assay in control MCF7 (A) and PC3 cells (B) or in cells transiently transfected with SMAR1. Images represent control cells and SMAR1 siRNA transfected cells at 0 hr and after 24 hr of transfection. (7.66 MB TIF) [file pone.0000660.s005.tif]
