# Supplementary material for: p53 Target Gene SMAR1 Is Dysregulated in Breast Cancer: Its Role in Cancer Cell Migration and Invasion
Source: PLoS One. 2007 Aug 1;2(8):e660. doi: 10.1371/journal.pone.0000660 (PMC1924604; doi:10.1371/journal.pone.0000660)
Supplement: Table S1 — Percent population shift towards G1/S and G2/M phase in Doxorubicin (0.5 µM) treated with and without siRNA (100 nM) compared to control untreated synchronized 293 cells. (0.03 MB DOC) [file pone.0000660.s006.doc]

**Table S1**

Table S1: Percent population shift observed towards G1/S and G_2_/M in 293 cells by Cell cycle analysis

| Time (hr) Doxorubicin Doxorubicin +siRNA |
| --- |
| G1/S G_2_/M G1/S G_2_/M |
| 0 - - - -  8 9 5 - -  16 13 17 4 -  24 15 2 9 -  36 18 22 16 -  48 15 23 8 30 |
